# Results of transoral laser microsurgery in 102 patients with squamous cell carcinoma of the tonsil

**DOI:** 10.1007/s00405-012-2335-6

**Published:** 2012-12-29

**Authors:** Martin Canis, Alexios Martin, Martina Kron, Alexandra Konstantinou, Friedrich Ihler, Hendrik A. Wolff, Christoph Matthias, Wolfgang Steiner

**Affiliations:** 1 Department of Otorhinolaryngology, Head and Neck Surgery, University of Göttingen, Robert-Koch-Str 40, 37099 Göttingen, Germany; 2 Department of Audiology and Phoniatrics, University of Berlin, Berlin, Germany; 3 Institute of Epidemiology and Medical Biometry, University of Ulm, Ulm, Germany; 4 Department of Radiation Oncology, University of Göttingen, Göttingen, Germany

**Keywords:** Transoral laser microsurgery, Carcinoma of the tonsil, Oncologic results, Functional outcomes

## Abstract

The objective of this study is to assess the feasibility of transoral laser microsurgery (TLM) in the treatment of squamous cell cancer of the tonsil and to report the oncological and functional outcomes, using retrospective chart review in the setting of single-institute, academic tertiary referral center. Between October 1987 and December 2006, 102 patients were eligible for this study, mostly suffering from advanced disease: 13 % presented with stage I and II (UICC/AJCC 2002) tumors and 87 % with stages III and IVa. The median follow-up was 63 months. All patients were treated by TLM with (or without) neck dissection (95 %) and with (66 %) postoperative radiotherapy. Overall survival, recurrence-free survival, disease-free survival, local control and loco-regional control were analyzed as end points. Rate of tracheotomies, postoperative complications and swallowing function were also analyzed. 5-year Kaplan–Meier local and loco-regional control was 78 % for pT1 and pT2 and 75 % for pT3 and pT4a tumors. 5-year Kaplan–Meier disease-free survival, recurrence-free survival, and overall survival and was 74, 64 and 59 % for stage I and II, 68, 60 and 56 % for stage III and IVa, respectively. Our data supports the conclusion, that TLM should be considered as a therapeutic option for the treatment of cancer of the tonsil. The oncological and functional results are comparable to any other treatment regimen, while the morbidity and complications tend to be lower.

## Introduction

Squamous cell carcinoma (SCC) of the tonsil is one of the most common types of cancer in the oropharynx worldwide. Within the United States it accounts for over 12 % of all head and neck cancers and in most cases is diagnosed at an advanced stage [1]. Where the incidence reaches 20 %, tonsillar carcinoma is the most significant subgroup of oropharyngeal cancer [2]. Incidence of oropharyngeal cancer strongly correlates to the consumption of alcohol and tobacco [3]. Furthermore, the incidence of carcinoma of the tonsil has significantly increased over the last 10 years, particularly in a younger age group, as a consequence of human papilloma virus (HPV) [4].

The available treatment options for tonsillar cancer are open surgery, endoscopic transoral surgical approaches and non-surgical procedures. Depending on the tumor size and the defect following tumor resection, open surgical procedures can range from a simple resection with primary closure to local or regional flaps and finally to microvascular free flaps. However, postoperative loss of function, especially swallowing, is directly associated to the extent of resection and reconstruction [5]. In addition, surgery in most cases is followed by adjuvant radiotherapy, which adds to the postoperative morbidity. Radical surgical procedures have not been proved to increase overall survival of the patients and therefore other therapeutic options have gained importance.

Primary radiotherapy, which can be combined with simultaneous chemotherapy, or induction chemotherapy offers a non-surgical option with salvage surgery for any failure. This organ preserving non-surgical approach is widely favored, especially in the treatment of advanced disease, as oncological results are reported to be comparable to those achieved by open surgery with reconstruction, while morbidity and severe or fatal complications are reported to be lower [6–9]. However, many patients undergoing this treatment develop severe swallowing disturbances including aspiration pneumonia. High-dose chemoradiotherapy protocols have high rates of acute and late toxicity affecting the upper aero-digestive tract function with notable rates of morbidity and mortality.

Transoral laser microsurgery (TLM) is a surgical alternative for all tumor categories of the upper aero-digestive tract [10–13] and was introduced by Steiner [10] in the early eighties. It provides the surgeon with a procedure that has the advantage of accurate tumor resection under high microscopic magnification without the need for reconstruction procedures. For early stage carcinoma, the minimum excision should be at least a tonsillectomy, and usually an excellent exposure can be achieved when treating tonsillar cancer. The patient is placed in a low Trendelenburg position, and the region of interest is exposed with appropriate gags and tongue depressors [14]. The Steiner distending oro-pharyngoscope can be used to gain a better view and access to tonsil carcinomas infiltrating the base of tongue and the vallecula.

## Patients and methods

The majority of patients presenting with carcinoma of the tonsil were treated exclusively by TLM with a transoral approach. The exceptions were those patients with very advanced tumors with extension to the carotid artery or to the skull base. These patients cannot be safely treated by an entirely endoscopic approach and required a combined transoral and open transcervical approach. Previously untreated tumors were staged according to the current classification of the Union for International Cancer Control (UICC) and the American Joint Committee on Cancer (AJCC) [15].

Exclusion criteria for this study were non-SCC tumors, simultaneous second primary tumor patients, those with simultaneous distant metastases, and N3 neck disease.

This retrospective study comprised 102 patients which presented with SCC of the tonsil (T1–4, N0–2, M0) and which were treated (October 1987–December 2006) by TLM with curative intent. During the time of the presented patient group (1987–2007), the importance of HPV and the surrogate marker p16 had not fully come to light and therefore has not been evaluated routinely.

Of these patients, 82 were male (80 %) and 20 were female (20 %). The median age was 57 years, with a range from 30 to 82 years. All patients were followed for at least 24 months, the median follow-up period being 63 months. Stage distribution was as follows: stage I, 3 patients (3 %), stage II, 10 patients(10 %), stage III, 29 patients (28 %) and stage Iva, 60 patients (59 %). 22 patients presented with a pT1 tumor category (22 %), 24 with pT2 (24 %), 42 with pT3 (41 %) and 14 with pT4a (13 %).

Pretherapeutic assessment consisted of a microscopic and rigid or flexible endoscopic examination in the outpatient clinics, followed by sonographic examination of the neck. Computed tomography (CT) or magnetic resonance imaging (MRI) of the neck was undertaken unless the patient presented with satisfactory imaging performed at the referring hospital. Further, standard preoperative investigations included X-ray examination of the chest and ultrasonography of the abdomen.

Panendoscopy was performed under general anesthesia at the beginning of the surgical procedure to exclude any second primary tumor in the aero and upper digestive tract. In contrast to the classic en bloc resection, the tumor was then resected in a step by step procedure under microscopic magnification using a CO_2_ laser (40c, Lumenis, Dreieich, Germany) in continuous, superpulse mode [14], average power 6 W. Using this technique the surgeon follows and completely excises the tumor, while preserving as much healthy and functionally important tissue as possible. The cutting characteristics of the CO_2_ laser within the tissue help the surgeon to differentiate between healthy tissue and tumor under the high magnification microscopic view. In the case of tonsillar cancer, the lymphatic tissue structure and any associated chronic inflammatory reaction within the tonsils may make this assessment more difficult [14]. When following the tumor laterally, high magnification and low CO_2_ laser power settings should be used because of the proximity to the large vessels. If a safe, complete resection via the transoral approach cannot be achieved, an external approach should be undertaken or combined with the TLM.

Tumors were resected under the microscope with surgical resection margins in healthy tissue of at least 5–10 mm. If the histopathological analysis (frozen section) of the resected specimen showed a positive resection margin, an additional resection was carried out to obtain a clear margin.

87 % of these patients underwent a selective neck dissection of the levels II and III because they presented with: (1) advanced primary disease or (2) the tumor infiltration depth was >3 mm at transoral resection, or (3) preoperative imaging revealed suspicious lymph nodes Patients underwent bilateral neck dissection if imaging revealed suspicious lymph nodes bilaterally, or if suspicious lymph nodes were seen only on one side, but no adjuvant (chemo)radiotherapy was planned. This elective selective neck dissection was usually performed 8–10 days after resection of the primary. This regime and time scale have the substantial advantage of avoiding any fistulas and add the possibility to do a re-resection in cases where postoperative histopathology shows that clear margins were not reached during the first TLM procedure.

Adjuvant radio(chemo-)therapy was administered in cases of pN2 neck disease (pN2a, pN2b) or when the histopathological examination revealed extracapsular spread or lymphangiosis carcinomatosa. Patients were followed up with a regular interval on an outpatient basis. Examination was undertaken of the oral cavity and oropharynx under microscopic magnification, palpation of the resection area, a rigid 90° or 70° endoscopy of the larynx and the lower parts of the pharynx as well as a ultrasonographic examination of the neck.

All survival rates were calculated with a 95 % confidence interval using the Kaplan–Meier method. The main points assessed as end points were overall survival, recurrence-free survival, disease-specific survival, local and loco-regional control, distant metastases and second primaries. Overall survival was calculated on deaths from all possible causes and disease-specific survival on deaths from tonsil cancer and deaths from therapy. For the purposes of the statistics, recurrence-free survival, intercurrent deaths, deaths due to secondary primary tumors, and patients alive without recurrences were regarded as censored observations. Events included local and/or regional recurrences, distant metastases and deaths due to disease. For the calculation of local control rate, only local recurrences were considered as events, while patients alive without local recurrence or death regardless of reason were counted as censored. The definition of local recurrence included carcinoma in situ as well as a carcinoma occurring after completion of primary treatment. All events were measured from the day of surgery to the date of their occurrence or the date of the last follow-up. For all statistical analyses, a *p* value of less than 0.05 was considered significant.

## Results

### Patients and therapy

One hundred and two patients were eligible for this study, with age ranging from 30 to 82 years, a median age of 57 years, and a median follow-up period of 63 months. All 102 patients were staged according to the UICC/AJCC [15] classification, exact pT/pN-category distribution is detailed in Table 1.

**Table 1:** pT/pN-category distribution

	cN0	pN0	pN1	pN2a	pN2b	pN2c	Total
pT1	0	3	7	4	8	0	22
pT2	2	8	3	0	11	0	24
pT3	3	14	5	1	19	3	42
pT4a	0	3	1	1	7	2	14
Total		30	16	6	45	5	102

3 patients (3 %) were exclusively treated with transoral laser surgery, 32 (31 %) with laser surgery and neck dissection, 65 (64 %) with laser surgery, neck dissection and adjuvant (chemo)radiotherapy and two patients (2 %) with laser surgery and adjuvant (chemo)radiotherapy. Neck dissection was performed unilaterally in 78 cases (80 %) and bilaterally in 19 cases (20 %). Most (87 %) were selective neck dissections of levels II and III. 11 of 97 (11 %) were treated by modified radical neck dissection. Adjuvant radio(chemo-)therapy was administered in 67 patients (66 %).

Adjuvant (chemo)radiotherapy was mainly performed in cases of advanced neck disease (N2a/b/c) or when the histopathological examination revealed extracapsular spread and/or lymphatic micrometastases. From 10/1987 to 12/1994 (23 patients), the (chemo)radiotherapy schedule was composed of two fractions per day, separated by 6-h intervals. Each fraction consisted of 210 cGy (1.25 MV ^60^CO) preceded by a dose of 50 mg/m^2^ carboplatin i.v. daily. A total radiation dose of 5,670 cGy was applied to the neck and the primary tumor over 6 weeks as split-course regimen. A break of 2 weeks was planned between the 2 last weeks of treatment. Treatment was given 4 days a week. *Cis*platinum-based chemotherapy was given concomitantly in 11 cases.

From 01/1995 to 12/2004 (38 patients), normofractionated radiotherapy (2 Gy/d, 5 times/week) was delivered as follows: parallel, opposed lateral portals were applied, matched to a single anterior portal encompassing the primary tumor and associated nodal drainage sites up to a maximum dose of 50 Gy. Finally, a 3D conformal external beam radiotherapy technique was used to boost the total dose to 60 Gy, including the primary tumor and involved lymph nodes. The spinal cord was limited to a maximum of 45 Gy. Concomitant chemotherapy was applied in seven cases.

From 12/2004 to 12/2006 (3 patients) normofractionated (2 Gy/d, five times/week) 3D conformal external beam radiotherapy was given with concomitant *Cis*platinum-based chemotherapy. The primary tumor and involved lymph nodes and potential drainage sites on both sides of the neck, including the supraclavicular region, were covered with 50 Gy in a first phase followed by a boost up to a total dose of 64 Gy, including the primary tumor and involved lymph nodes. The spinal cord was limited to a maximum of 45 Gy.

### Oncological results

**Fig. 1:**
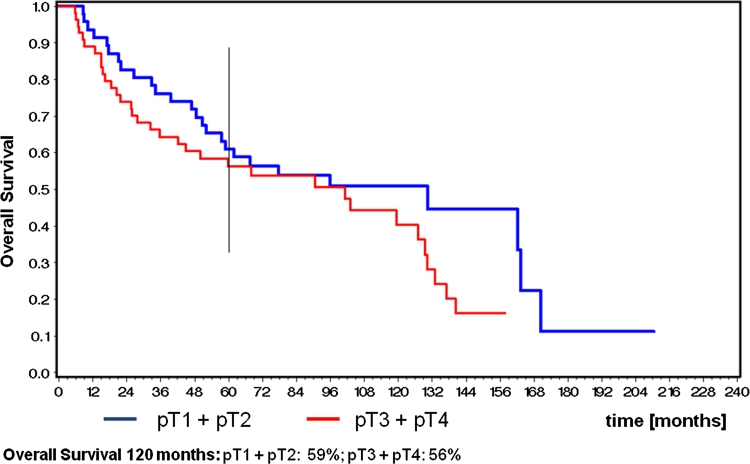
Kaplan–Meier estimates for 10-year overall survival

**Fig. 2:**
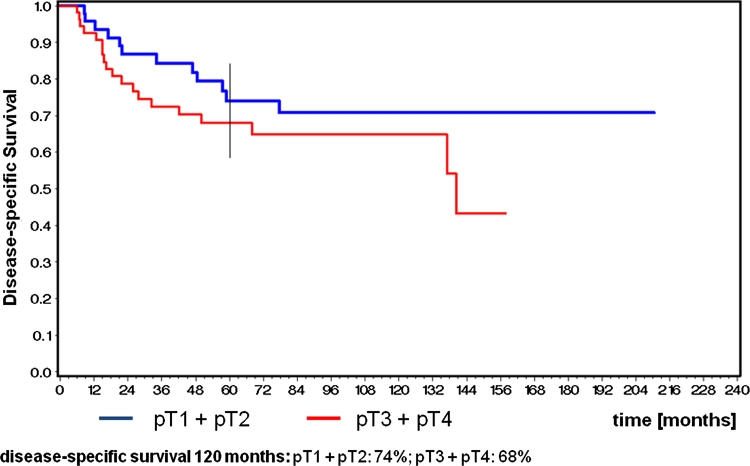
Kaplan–Meier estimates for 10-year disease-specific survival

**Fig. 3:**
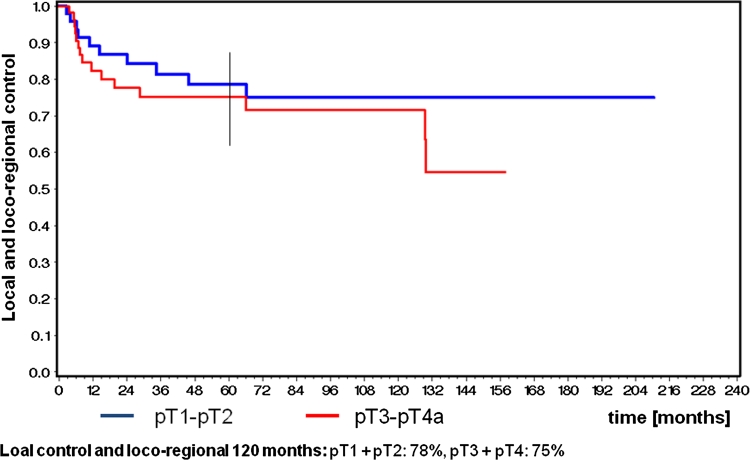
Kaplan–Meier estimates for 10-year local control

Because of the relatively small numbers of patients, stages I and II were combined for statistical analysis. The 5-year Kaplan–Meier estimates for overall survival were 59 % for pT1 and pT2, 56 % for pT3 and pT4 **(**Fig. 1). The corresponding figures for 5-year recurrence-free survival were 64 % for stages pT1 and pT2 and 60 % for pT3 and pT4. Disease-specific survival was measured as 74 % for pT1 and pT2 and 68 % for pT3 and pT4 **(**Fig. 2). Local control and loco-regional control were combined for statistical analysis. 5-year local control and loco-regional control rates were 78 % for pT1 and pT2 and 75 % for pT3 and pT4a tumors **(**Fig. 3).

At the time of this analysis, 42 patients were alive with no evidence of disease (41 %), 31 patients (30 %) had died TNM-related, 10 patients died from second primary tumors (10 %) and another 19 (19 %) died of intercurrent disease. 18 (18 %) local and 7 (7 %) loco-regional failures occurred, while four patients (4 %) had late and six (6 %) recurrent neck metastases. An incidence of 21 second primaries (21 %) was observed, among which ten occurred in the head and neck region, five in the lung, four in the gastro-intestinal tract and two in the prostate. Distant metastases without simultaneous local, loco-regional or regional recurrence were observed in six cases (6 %). Only 8 of the 32 patients with local, loco-regional or regional failure could be successfully salvaged.

### Complications and functional results

The main complication was postoperative bleeding, which occurred 18 times. Fourteen hemorrhages (15 %) originated postoperatively in the oropharynx and were managed by a transoral microscopic approach with coagulation or clipping. Four hemorrhages (4 %) occurred postoperatively in the neck after neck dissection and were treated by neck revision. One patient sustained significant temporary laryngeal edema postoperatively, which was successfully treated conservatively. One temporary tracheotomy due to aspiration but no permanent tracheotomy was necessary as part of the treatment in all 102 cases. The median duration of nasogastric feeding tube placement was 10 days in 64 patients (63 %), with a minimum of 2 days and a maximum of 129 days. In this group, the nasogastric tube had to be replaced by gastrostomy tubes in four patients (4 %) because of swallowing difficulties, two of them permanently. Another six patients required gastrostomy tubes after adjuvant radiotherapy, one permanently. No patient died of treatment related causes.

## Discussion

Due to the lack of prospective randomized trials and site-specific analyses, currently there is no consensus regarding management of tonsillar squamous cell carcinomas.

Although several retrospective investigations have been published, discussion of the oncologic outcomes is still controversial, since evaluation of evidence supporting the effectiveness of one treatment over the other is complicated by a very heterogenous group of cancers (tonsil vs. oropharynx), variable T/N categories, different outcome measures and statistics.

For the treatment of tonsillar carcinomas, different surgical techniques have been described including conventional surgery [16], TLM [10], transoral robotic surgery [17] and transoral lateral oropharyngectomy [18]. All these approaches have its inherent advantages and disadvantages. In the past, however, surgical intervention often has been open en bloc resection to the pharynx with radical mandibular osteotomy after lip lysis including tracheotomy and radical neck dissection. In contrast, TLM is a minimally invasive surgical technique that combines radical oncological treatment with relatively rapid recovery and low side effects. Since the introduction of TLM for the treatment of limited head and neck malignancies [19], indications for its use have been vastly expanded to all tumor categories of the upper aero-digestive tract [10–13] by Steiner in the early eighties.

Recent studies [20–22] have demonstrated that TLM in combination with adjuvant treatment provides excellent survival and swallowing proficiency for early and advanced tonsillar squamous cell carcinomas. Haughey et al. [21] investigated 204 patients with stage III and IV oropharyngeal carcinoma. Ninety-eight patients presented with tonsillar cancer and were primarily treated with TLM in combination with or without neck dissection and adjuvant therapy. 5-year overall, disease-specific and disease-free survival rates of all patients were 78, 84 and 74 %. Corroborating the value of TLM in treatment of tonsillar cancer, Grant et al. [20] observed comparable results in 69 patients with T1–T3 oropharyngeal cancer. Twenty-eight patients presented with tonsillar carcinoma. All patients were primarily treated with TLM in combination with uni- or bilateral neck dissection (83 %) and adjuvant radiotherapy (36 %). For stage I, II, and III disease, the 5-year Kaplan–Meier estimates of loco-regional control were 90, 73, and 70 %, respectively. The 5-year overall survival estimate was 86 %.

In the present study, the achieved 5-year overall survival estimates were 59 % for pT1 and pT2 tumors and 56 % for category pT3 and pT4. Five-year local control and loco-regional control rates were 78 % for pT1 and pT2 and 75 % for pT3 and pT4a tumors. However, as indicated at the beginning data are fairly comparable since results of diverse studies originate from a very heterogenous group of cancers.

While the oncological results are comparable to more radical interventions, a major advantage of TLM is the relatively low morbidity. This is made possible by the transoral approach under high microscopic magnification with preservation of functionally important structures and avoidance of dismantling the musculoskeletal infrastructure of the oral cavity and the pharynx. Recently, Rich et al. [23] investigated swallowing function after TLM with or without neck dissection and adjuvant therapy for 58 patients with advanced tonsillar cancer. Longitudinal analysis of swallowing revealed that 1 month postoperatively 82 % had good swallowing. At 3 months postoperatively, which coincided with the administration of adjuvant therapy, the percentage of patients with good swallowing dropped to 55 % and rose to 89 % by the end of 12 months.

Comparably, Haughey et al. [21] observed the prevalence of gastrostomy tubes after TLM as primary treatment in patients with advanced stage III/IV oropharyngeal cancer as 18.8 % after 1 year, 9.3 % after 2 years and 3.4 % after 3 years. In line with this data, Grant et al. [20] investigated gastrostomy tube use after TLM in 69 patients with oropharyngeal caner (tonsil *n* = 28). No patient needed a permanent gastrostomy tube.

In our series, the median duration of nasogastric feeding tube placement was 10 days in 64 patients (63 %) and had to be replaced by gastrostomy tubes in four patients (4 %) because of swallowing difficulties, two of them permanently. In both patients, advanced tumor resections including parts of the tongue base and the hypopharynx had to be performed. Another six patients required gastrostomy tubes during adjuvant radiotherapy, one permanently. Therefore, in cases of postoperative swallowing impairment and planned adjuvant treatment, temporary placement of a gastrostomy tube may be considered to improve nutrition during radiotherapy. However, this decision has to be taken individually for each case by the surgeon and the patient. Even evaluation of function is not only a matter of whether a patient is still on a nasogastric or gastrostomy tube, it may serve as indicator for functional outcomes and corroborates the value of TLM as primary therapy of tonsillar cancer.

In our series, we experienced 18 postoperative bleedings that were managed without tracheotomy. In one case of aspiration, a temporary tracheotomy had to be performed, no permanent tracheotomy was needed. In recent literature using TLM for resection of oropharyngeal cancer, rates of tracheotomy depend on T-category and range between 0.1 % [20] and 16 % [21]. However, the decision for or against tracheotomy has to be taken individually by the surgeon under consideration of the medical demands and its own demands for safety.

TLM is also an appropriate approach for patients who require microvascular free tissue transfer because of large vessel exposure or extended pharyngeal or palatine defects. In addition, for resection of T3–T4 tumors large wounds are created which may lead to severe scar tissue formation and if the pterygoid muscles are involved also trismus might occur. In these situations significant functional recovery with free flap reconstruction is possible and necessary [24]. However, in our retrospective case series, no free tissue transfer was needed since functional outcomes after primary healing were satisfying.

Cervical lymph node metastasis is an important prognostic factor in patients with tonsillar cancer since nodal disease significantly decreases survival rates [25]. Therefore, neck dissection is a useful procedure in terms of staging and for the indication of further adjuvant treatment. Patients with occult nodal disease may be identified or confirmed to be pN0. However, there is controversy with regard to the elective neck treatment of contralateral neck nodes. Chung et al. [26] investigated 76 cases with tonsillar cancer (81.6 % stages III–IV) and found an overall contralateral metastasis rate of 40.9 %. Lim et al. [27] analyzed 43 patients with pT1–pT4 tonsillar cancer and found contralateral metastasis in 16 % of all patients. Recently, Mantsopoulos et al. [28] investigated the surgical treatment of locally limited pT1 and pT2 tonsillar carcinomas. In 202 patients a bilateral neck dissection was carried out in 77 cases, and contralateral neck metastases were found in 11.7 % (9 cases). However, in the majority of cases patients presented already with clinically positive contralateral lymph nodes. In cases with only positive ipsilateral side, incidence of contralateral metastasis was only 3 %. In our cohort, 55 % of the patients presented with advanced pT3–pT4 tumors. Selective neck dissection was performed unilaterally in 78 cases (80 %) and bilaterally in 19 cases (20 %). In five cases (5 %) of advanced disease, bilaterally positive lymph nodes were found. In line with Mantsopoulos et al. [28], dissection of the contralateral neck side in locally limited tonsillar tumors may be reserved for cN2c status.

Survival and functional outcomes of primary chemoradiation have never been tested against surgical approaches, especially TLM, in randomized controlled trials. Results from retrospective case series using chemoradiation compare not favorably but show significant acute and chronic toxicity due to high-dose radiotherapy often sensitized to an even greater functional impact by concurrent chemotherapy. Nonetheless, chemoradiation has been promoted as treatment of choice for oropharyngeal and tonsillar carcinomas. Mendenhall et al. [7] reported 503 tonsillar cancers, most of them stages III and IV, treated with radiotherapy (RT) alone (61 %) or with RT and salvage neck dissection (39 %). Five-year overall survival is reported as 54 % for stage I, 61 % for stage II, 62 % for stage III and 57 % for stage IVa. These rates are comparable with our own findings. However, treatment protocols show high rates of acute and chronic toxicity [29, 30] such as swallowing dysfunction. Perez et al. [31] investigated 384 patients presenting with all stages of tonsillar carcinoma (80 % T3–T4) which were treated with RT alone (*n* = 154), with irradiation followed by surgery (*n* = 144) and with adjuvant irradiation (*n* = 86). In patients with advanced T3–T4 tumors, surgery in combination with adjuvant treatment offered better tumor control than single-modality or preoperative irradiation procedures. However, at this point of time, no concurrent chemotherapy was offered. In line with these results, Poulsen et al. [32] investigated 148 with curable stage III and stage IV squamous cell carcinoma of the tonsil. One hundred and two patients were treated with conventional surgery ± adjuvant (chemo-)radiotherapy, 46 patients were treated with primary (chemo)radiation. Analyses showed that patients after surgery ± (chemo-)radiotherapy had a significantly superior overall survival (57 vs. 41 %) and a trend towards better loco-regional control (88 5 vs. 73 %). In a retrospective chart review, Lamarre et al. [33] investigated 17 patients who underwent radical tonsillectomies with neck dissection ± (chemo-)radiotherapy, and 33 patients underwent (chemo)radiation-based treatments for T1 and T2 and N0 to N2a tonsil cancer. Five-year overall survival for patients undergoing primary surgery ± adjuvant treatment and primary radiation-based treatment did not show any statistical significant differences. However, treatment groups were small and thus may be underpowered. Johansen et al. [34] investigated a total of 289 patients with carcinoma of the oropharynx mainly (58 %) originating in the tonsil area. Only eight patients were treated with primary surgery, all others were treated by primary radiotherapy. The authors reported a recurrence of the primary tumor in 72 %, a 5-year loco-regional control, a disease-specific control and an overall survival of 38, 44 and 31 % which is inferior to our results. In early stage tonsillar disease, brachytherapy can achieve high local control and overall survival rates, the 5-year estimates being 98 and 76 % respectively with limited soft-tissue toxicity and no reported xerostomia or osteonecrosis [35]. The patients in this series were selected according to the therapeutic regimen, with 41 of 44 T1 and T2 tumors having no neck disease (N0). Good local control (100 % 2-year estimate) was achieved in a series of 24 patients treated using targeted intra-arterial chemoradiation according to the RADPLAT protocol, though a high rate of acute toxicity and complications was noted. Fifty percent of the patients developed grade 2 or 3 mucositis and three patients died during treatment [36].

To reduce xerostomia and other local side effects in our patients, only ipsilateral radiotherapy was used in cancers that did not cross the midline. This is in line with results reported by Jackson et al. [37] and Chronowski et al. [38] that point to a reduction of acute and late side effects, especially xerostomia, by sparing the contralateral parotid gland, while achieving oncological results comparable to standard radiotherapy. Using intensity modulated radiotherapy (IMRT) could be a way to reduce late dysphagia after such intensive chemoradiation, by partially sparing the pharyngeal constrictor muscles, which seem to be one reason for the mentioned swallowing problems [39].

In conclusion patients treated by TLM for squamous cell cancer of the tonsil have a low peri-/postoperative morbidity and complication rate. Only 2 % suffered from therapy-associated swallowing problems that required a permanent gastrostomy tube. No permanent tracheotomy was necessary in our patients. Our results and data from the literature show that oncological results achieved with TLM are at least comparable to any other current surgical and non-surgical treatment regimen. It is our opinion that TLM in combination with or without neck dissection and adjuvant therapy is an efficient option in the treatment of tonsil cancer. As these results originate from a single institution and a relatively small sample, they should be validated by multicenter prospective clinical trials.
